# Information Filtering via Biased Random Walk on Coupled Social Network

**DOI:** 10.1155/2014/829137

**Published:** 2014-07-22

**Authors:** Da-Cheng Nie, Zi-Ke Zhang, Qiang Dong, Chongjing Sun, Yan Fu

**Affiliations:** ^1^Web Sciences Center, School of Computer Science & Engineering, University of Electronic Science and Technology of China, Chengdu 610054, China; ^2^Institute of Information Economy, Hangzhou Normal University, Hangzhou 311121, China; ^3^Alibaba Research Center for Complexity Sciences, Hangzhou Normal University, Hangzhou 311121, China

## Abstract

The recommender systems have advanced a great deal in the past two decades. However, most researchers focus their attentions on mining the similarities among users or objects in recommender systems and overlook the social influence which plays an important role in users' purchase process. In this paper, we design a biased random walk algorithm on coupled social networks which gives recommendation results based on both social interests and users' preference. Numerical analyses on two real data sets, *Epinions* and *Friendfeed*, demonstrate the improvement of recommendation performance by taking social interests into account, and experimental results show that our algorithm can alleviate the user cold-start problem more effectively compared with the mass diffusion and user-based collaborative filtering methods.

## 1. Introduction

In the past two decades, the Web 2.0 and its applications have greatly accelerated the development of the Internet. They bring our lives much convenience as well as overwhelm us with too many resources in the information ocean. One typical scenario is online shopping in our daily life. When we are confronted with millions of books on http://www.Amazon.com or billions of different kinds of commodities on http://www.Taobao.com, indeed, it is very difficult to choose the relevant ones from countless candidates. This is the so-called* Information Overload* problem [1]. Therefore, an automatic way that can help us make the right decision under the* Information Overload* is a significant issue for both academic and industrial communities.

Search engines provide a way to help users find the useful information, which alleviates this dilemma partially: a user inputs the keywords and then the search engine returns the results accordingly. However, if different users input the same keywords, the search engine will return the same results. Besides, when users resort to a search engine, they must know how to clearly describe what they want by the keywords. But in most situations, users do not know what they really want or it is hard for them to find appropriate keywords. In this case, the recommender systems [2] have been designed to solve this problem.

Recently, social networks (SN) [3, 4] have become a powerful tool to characterize social relationship in online social services, emerging with various Web 2.0 applications [5] in evolutionary games [6, 7], community detection [8], medical science [9], and so forth. By taking advantage of social relationship in recommender systems, many traditional challenges can be partially solved, such as the cold-start problem [10] and data sparsity problem [11]. However, most researches are focused on mining the similarities among users or objects in recommender systems, and the social influence is seldom taken into account.

**Figure 1:**
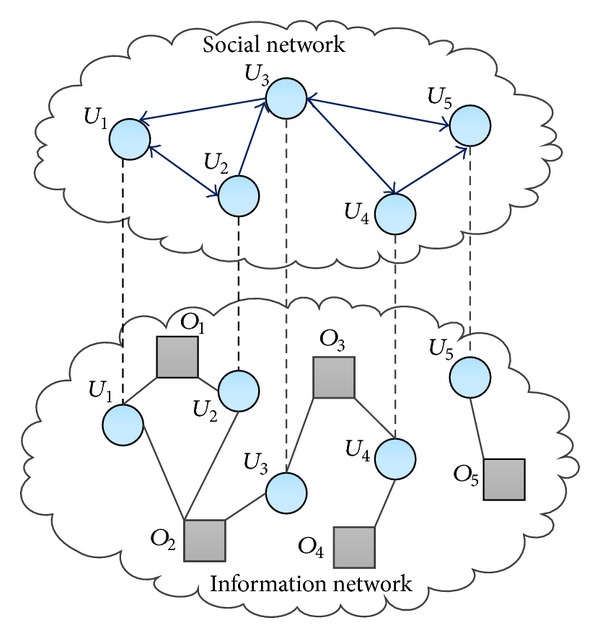
Illustration of a coupled social network with five users and five objects, where circles denoteusers and squares represent objects (color online). The social network (upper layer) consists of five users; the information network (lower layer) consists of five objects and five users, while user nodes are the same in the social network.

Coupled networks (CN), also known as interdependent networks [12], are usually composed of two layers of networks [12, 13], such as electricity/internet networks [14] and airport/railway networks [15]. Being similar with interdependent networks, a coupled social network (CSN) also contains the coupling nodes (users), which form a leader-follower relationship in the layer of social network and collecting relationship in the layer of information network.  Figure 1 gives an illustration of a simple CSN with five users and five objects, where circles denote users and squares represent objects; the social network (upper layer) consists of five users and the information network (lower layer) consists of five objects and five users, where the users are the same as those in the social network. It can be seen that *O*_5_ will not be recommended to *U*_4_ in the user-object network because only *U*_5_ collects object *O*_5_ and the value of similarity between *U*_4_ and *U*_5_ is zero. However, *U*_4_ follows *U*_5_ in the social network, which indicates that *U*_4_ may have similar interests with *U*_5_ to some extent; thus, we can accordingly recommend *O*_5_ to *U*_4_ via social network. Therefore, by making use of the social relationship between users, the user cold-start problem can be partially solved. When a new user comes to the system, we can recommend him/her some objects through the social network.

Moreover, most researchers focus their attention on mining the similarities among users or objects in recommender systems, and many researchers use the social interest to filter the recommendations, but we use the social interest to supplement the recommendations instead of filtering them. To our knowledge, the random walk algorithm on coupled social network remains yet to be investigated in recommender systems.

The contributions of this paper can be summarized as follows. (1) We use the social interest to supplement the recommendations instead of filtering them and we obtain more accurate recommendations. (2) We first propose a biased random walk recommendation algorithm on coupled social network, which considers the social interests as well as users' preference in the recommender systems. This method can improve the performance of recommendations. (3) Compared with the mass diffusion (MD) [16, 17] and user-based CF (UCF) [18, 19] methods, the proposed algorithm can alleviate the user cold-start problem more effectively.

This paper is organized as follows. We introduce the related works in Section 2. In Section 3, we propose a biased random walk algorithm on coupled social network. In Section 4 we describe the data sets and metrics used in this paper. We evaluate the performance of the proposed method in Section 5. Finally, we summarize this paper in Section 6.

## 2. Related Works

Collaborative filtering (CF) [18–25] is the most frequently used technology in recommender systems, which uses the collection history of users for mining the potential objects of interest to the target user. However, the CF algorithm only takes the similar users or objects into account and will lead to the same recommendation results to diverse users; namely, it is not conducive to the personalized recommendation. Meanwhile, the CF algorithm cannot deal with the cold-start problem [10]; that is, when a new user or object is added to the system, it is difficult to obtain recommendation or to be recommended because of lack of enough information. To alleviate this problem, many methods have been proposed, such as content-based [26], trust-aware [27, 28], social-impact [29], and tag-aware [30] methods.

Random walk [31] is a mathematical formalization of a path that consists of a succession of random edges, which is successfully used in recommender systems based on bipartite network [16, 32], namely, mass diffusion (MD for short) method [16]. Accordingly, many methods based on mass diffusion were proposed [17, 33]. Furthermore, random walk was successfully used in many fields, such as social network [34] and Top-*k* search [35]. However, there is a lack of study of random walk on coupled social network in recommender systems.

Massa and Avesani [36] proposed a social propagation method that is based on users' distance from a fixed propagation horizon, which increased the coverage of recommender systems. Esslimani et al. [37] proposed a feedback effect between similarity and social influence in online communities. By utilizing the social relations, we can obtain the strength of social relationship between users, and we can use this social relationship to generate more accurate recommendation results. Meanwhile, the literature [36, 38] demonstrated that recommendation performance can be improved by taking into consideration the effect of social network, and the methods are both filtering the useless information by social relationship.

Lai et al. [39] proposed a hybrid personal trust model which adaptively combines the rating-based trust model and explicit trust metric to resolve the drawback caused by insufficient past rating records. Community-based recommender systems have attracted much research attention; the authors [40] proposed a novel community-based framework that employs PLSA-based model incorporating social activeness and dynamic interest to discover communities. Wei et al. [41] proposed a multicollaborative filtering trust network algorithm, an improved version of CF algorithm designed to work on Web 2.0 platform, which can improve the prediction accuracy compared with the original CF algorithm. We believe that if the social relationship can be used to supplement the user-object network like the aforementioned example of Figure 1, we will get more accurate recommendations and alleviate the user cold-start problem. Motivated by this, we proposed a biased random walk (diffusion-based) method on coupled social network to generate recommendations. Therefore, new users can obtain recommendations as long as they are connected to others in social networks.

## 3. Method

In this section, we introduce the approach of diffusion on coupled social networks. Generally, a recommender system consists of two sets, *U* = {*U*_1_, *U*_2_,…, *U*_*m*_} and *O* = {*O*_1_, *O*_2_,…, *O*_*n*_} representing the *m* users and *n* objects, respectively. Denote *A*_*m*×*n*_ by the adjacent matrix of the user-object bipartite network, of which each element *a*_*iα*_ = 1, if user *U*_*i*_ has collected object *O*_*α*_, and *a*_*iα*_ = 0 otherwise. Analogously, denote *B*_*m*×*m*_ by the nonsymmetric adjacent matrix of user-user directed social network, of which each element *b*_*ij*_ = 1, if the user *U*_*i*_ has linked to user *U*_*j*_, and *b*_*ij*_ = 0 otherwise.

*Random Walk on Social Network*. Let *P*′ be the *m* × *m* transition probability matrix of a directed social network. The probability that a random walker at user *U*_*i*_ goes to user *U*_*j*_ on social network can be described as

(1) pij′={bijkiout,if  kiout≠00,otherwise,

where *k*_*i*_^out^ is the out-degree in social network, that is, the number of leaders of user *U*_*i*_. Denote *s*_*i*_′(*t*) by the probability from other users to user *U*_*i*_ at time *t*. Therefore, we have

(2) si′(t+1)={∑j=1mbijkioutsj′(t),if  kiout≠00,otherwise.

The initial probability for target user *U*_*i*_ is given by *s*_*i*_′(0) = 1, and *s*_*j*_′(0) = 0 for all of the other user *U*_*j*_. Thus, we can obtain the probability that a random walker goes from the target user to all other users at time *t*. 

*Random Walk on Bipartite Network*. Let *P*′′ be the *m* × *n* transition probability matrix of a bipartite network. The probability that a random walker at user *U*_*i*_ goes to object *O*_*α*_ on bipartite network can be described as

(3) piα′′={aiαki,if  ki≠00,otherwise,

where *k*_*i*_ denotes the number of collected objects of user *U*_*i*_, and the probability that a random walker at object *O*_*α*_ goes to user *U*_*j*_ on bipartite network can be described as

(4) pαj′′={ajαkα,if  kα≠00,otherwise,

where *k*_*α*_ denotes the number of users who have collected object *O*_*α*_ on bipartite network. Denote *s*_*i*_′′(*t*) and *s*_*α*_′′(*t*) by the probability of user *U*_*i*_ and object *O*_*α*_ on bipartite network at time *t*, respectively. Therefore, we have

(5) si′′(t+1)={∑α=1naiαkisα′′(t),if  ki≠00,otherwise,sα′′(t+1)={∑j=1majαkαsj′′(t),if  kα≠00,otherwise.

Similar to random walk on social network, the initial probability for target user *U*_*i*_ is given by *s*_*i*_′′(0) = 1. But the difference is the fact that there are two different nodes on bipartite network and the initial probability *s*_*j*_′′(0) = 0 and *s*_*α*_′′(0) = 0 for all the other user *U*_*j*_ and object *α*. In the odd time step and *t* ≥ 3, the probability of *s*_*α*_′′(*t*) means the probability of target user *U*_*i*_ selecting uncollected object *O*_*α*_. Therefore, we can obtain the recommendation list according to this probability for target user. 

*Biased Random Walk on Coupled Social Network*. Let *P* be the *M* × *M* transition probability matrix of a coupled social network, where *M* = *m* + *n*. In order to solve the user cold-start problem, suppose that a random walker at user *U*_*i*_ goes to their neighbors (leaders) on directed social network with probability *λ* ∈ (0,1), and to their neighbors on bipartite network with probability 1 − *λ*. What's more, a random walker at object *O*_*α*_ goes to all users who collect object *O*_*α*_ with equal probability. Thus, the target user finds the potential objects not only through other users with similar collecting interest on bipartite network, but also through their friends (leaders) on directed social network. Denote *s*_*i*_(*t*) and *s*_*α*_(*t*) by the probability of walker user *U*_*i*_ and object *O*_*α*_ on coupled social network at time *t*, respectively. Therefore, we have

(6) si(t+1)={λ·∑j=1mbijkioutsj(t)+(1−λ) ·∑α=1naiαkisα(t),if  ki≠0,  kiout≠0∑j=1mbijkioutsj(t),if  ki=0,  kiout≠0∑α=1naiαkisα(t),if  ki≠0,  kiout=00,otherwise,sα(t+1)={∑j=1majαkαsj(t),if  kα≠00,otherwise.

That is to say, initially, we assign the target user one unit of resource. Then *λ* (0 ≤ *λ* ≤ 1) proportion of the resource is evenly distributed to the user's social neighbors through the directed links (social network), and 1 − *λ* proportion is distributed to collected objects through the undirected links (bipartite network). In (6), when *k*_*i*_^out^ = 0 then *λ* = 0; it means that user *U*_*i*_ has no outlinks in social network; therefore, he/she will distribute all of his/her resources to bipartite network. Similarly, when *k*_*i*_ = 0, then *λ* = 1; user *U*_*i*_ will distribute all of his/her resources to social network. The initial score for target user *U*_*i*_ is given by *s*_*i*_(0) = 1, *s*_*j*_(0) = 0, and *s*_*α*_(0) = 0 for all the other user *U*_*j*_ and object *α*. Thus, we can obtain the recommendations by ranking the score *s*_*α*_ of all objects at time *t* for target user. At time *t* = 2, the recommendations are obtained only from social network, that is, his/her social leaders. At time *t* = 3 and *λ* = 0, the recommendations are obtained only from bipartite network and it is the pure MD algorithm.

Thus, the probability that a random walker arrives at the object at time *t* is recognized as the possibility that the target user purchases this object. We call this algorithm biased random walk (BRW). For the example in Figure 1, the transition probability matrix *P* for coupled social network is given in the following equation:

(7) P=U1U2U3U4U5O1O2O3O4O5U1U2U3U4U5O1O2O3O4O5(01/20001/41/40001/401/4001/41/40001/6001/61/601/41/40000001/2001/41/40001/20000001/21/21/2000000001/31/31/30000000001/21/200000000010000000000100000).

Consider *λ* = 0.5 and *t* = 2; then *P*(*U*_3_, *O*_5_) = 0.0833, which means users *U*_3_ and *O*_5_ are reachable within 2 steps with 0.0833 probability through the coupled social network. On the other hand, without social network, the random walk distance on the original bipartite network *P*′′(*U*_3_, *O*_5_) = 0 for an arbitrary time *t* because *U*_3_ and *O*_5_ are not reachable from each other in bipartite network.

## 4. Data and Metrics

### 4.1. Data Sets

To evaluate our algorithm's performance, two real data sets are analyzed in the experiments. The data sets are from http://www.epinions.com and http://www.friendfeed.com, both of which provided user-objects collecting information and user-user social relationship. The* Epinions* data set was collected by Paolo Massa in a 5-week crawl (November/December 2003) from the http://www.epinions.com website [36] and the Friendfeed data set was collected by Fabio Celli et al. from http://www.friendfeed.com (September 6, 2009 to September 19, 2009) [42]. We extract a smaller data set by randomly sampling the whole records of user activities in both* Epinions* and* Friendfeed* data sets. 4,066 users, 7,649 objects, 154,122 collected links, and 217,071 social links in total were found in the* Epinions* data set.* Friendfeed* contains 4,148 users who collected 5,700 objects, 96,942 collected links, and 386,804 social links.  Table 1 shows the basic statistics for two representative data sets. Denote |*U*|, |*O*|, and *N*_*R*_ by the number of users, objects, and ratings, respectively. Sparsity = *N*_*R*_/(|*U*| × |*O*|) denotes the data sparsity of user-objects network.

### 4.2. Metrics

To test our algorithm's performance, each information network is randomly divided into two parts: the training set consists of 90% entries and the remaining entries constitute the testing set. The training set is treated as known information used for generating recommendations, while the training set is regarded as unknown information used for testing the performance of the recommendation results. To evaluate the proposed algorithm, we employed five different metrics that characterize not only the accuracy of recommendations, but also the diversification, which are defined as follows.

(*1) Precision [43]*. Precision represents the probability that the selected objects appeared in the recommendation list which is shown as

(8) $$\begin{array}{c} {\text{Precision}}_{i}=\frac{N_{rs}^{i}}{L}, \end{array}$$

where Precision_*i*_ represents user *u*_*i*_'s precision, *N*_*rs*_^*i*^ denotes the number of recommended objects that appeared in the *U*_*i*_'s testing set, and *L* represents the length of recommendation list. By averaging over all users' precisions, we can obtain the whole recommender systems' precision as

(9) $$\begin{array}{c} \text{Precision}=\frac{1}{m}\overset{m}{\underset{i=1}{\sum }}{\text{Precision}}_{i}, \end{array}$$

where *m* represents the number of users. Obviously, a higher precision means a higher recommendation accuracy. 

(*2) Recall [43]*. Recall represents the probability that the recommended objects appeared in user's collected list shown as

(10) $$\begin{array}{c} {\text{Recall}}_{i}=\frac{N_{rs}^{i}}{N_{p}^{i}}, \end{array}$$

where Recall_*i*_ represents user *u*_*i*_'s recall and *N*_*p*_^*i*^ is the number of objects collected by user *u*_*i*_ in the testing set. Averaging over all individuals' recall, we can obtain the recall of the whole recommender system. 

(*3) F-Measure [43]*. Generally speaking, for each user, recall is sensitive to *L* and a larger *L* generally gives a higher recall but a lower precision. The* F-*measure, that assigns equal weight for precision and recall, is defined as

(11) $$\begin{array}{c} F\text{-measur}{\text{e}}_{i}=\frac{2\cdot {\text{precision}}_{i}\cdot {\text{recall}}_{i}}{{\text{precision}}_{i}+{\text{recall}}_{i}}. \end{array}$$

By averaging over all users' *F*-measure, we can also obtain the whole system's *F*-measure. 

(*4) HD [17]*. HD is a metric to measure the diversity of users' recommendation lists. It uses the Hamming distance to measure the difference of recommendation lists between users *u*_*i*_ and *u*_*j*_, which is defined as

(12) $$\begin{array}{c} \text{H}{\text{D}}_{ij}\left(L\right)=1-\frac{Q_{ij}\left(L\right)}{L}, \end{array}$$

where *Q*_*ij*_(*L*) is the number of commonly recommended objects shown in top-*L* locations of users *u*_*i*_ and *u*_*j*_'s recommendation list. Averaging over all pairs of users' HD_*ij*_(*L*), we can obtain the HD of the recommender algorithm. Obviously, higher HD means higher diversity of users. 

(*5) Ranking Score (r* *) [44]*. Generally, the recommender system aims to generate a ranking list for the target user's uncollected objects through the prediction score. In the recommender systems, one of the most used metrics to evaluate the algorithm's performance is ranking score, which measures the users' satisfaction of the ranking list, and is defined as follows:

(13) $$\begin{array}{c} r_{i\alpha }=\frac{L_{i\alpha }}{N_{i}}, \end{array}$$

where *L*_*iα*_ is the position of uncollected object *α* in user *U*_*i*_'s ranking list and *N*_*i*_ is the length of the user *U*_*i*_'s ranking list. By averaging all links' ranking score value we can obtain the whole system's ranking score value *r*. A small *r* means the recommender system puts the user's favorite objects in a top place in the recommender list; hence, the smaller *r* is, the better an algorithm's performance will be.

## 5. Results

**Figure 2:**
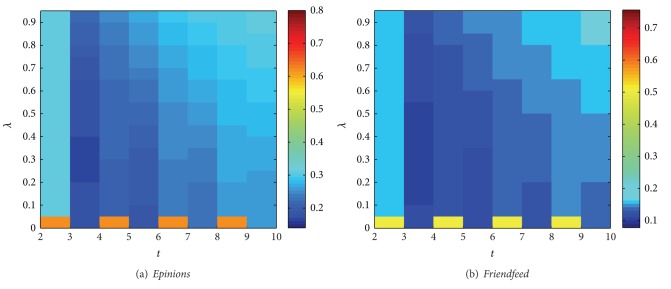
Ranking score values on* Epinions* and* Friendfeed* data sets (color online).

Figure 2 shows the ranking score values on* Epinions* and* Friendfeed* data sets. From the figure we can see that the best performance is achieved at time *t* = 3. At time *t* = 2, the recommendations are obtained only from social network and when *λ* = 0 it will generate random recommendation results since the ranking score value *r* is much bigger than others. When *λ* = 0 the resource will spread only on bipartite network; therefore objects get scores in odd time steps only, and user get scores in even time steps only. In addition, the ranking score will fluctuate up and down alternately with time *t*. That is because when *λ* > 0 the recommendations are obtained from social interest in odd time step, and from both social interests and collecting preferences in even time step. With the increase of time *t* in even and odd time step, respectively, the ranking score becomes worse due to the existence of the redundant correlations [45].

**Figure 3:**
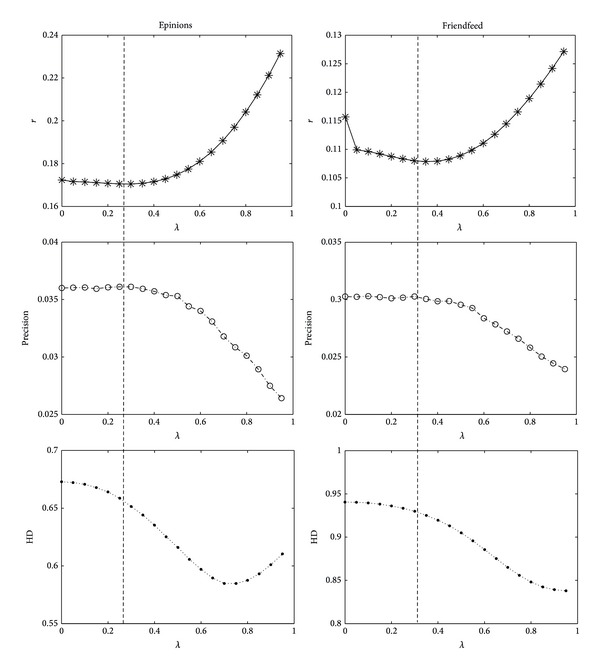
The precision and HD when recommendation list *L* = 20 and *r* in the* Epinions* and* Friendfeed* data sets. Each result is obtained by averaging over 10 independent runs, each of which corresponds on a random division of training set and testing set.

The best ranking score performance occurs at time *t* = 3; that is, when we consider the social interest in the recommender systems, it will improve the performance of recommender systems.  Figure 3 shows the experimental results of precision, recall,* F*-measure, HD with recommendation list *L* = 20, and ranking score *r* on* Epinions* and* Friendfeed* data sets at time *t* = 3. *λ* = 0 gives the pure MD algorithm. It can be found that when the parameter *λ* reaches the optimal value, the precision, recall, *f*-measure, and *r* almost simultaneously reach the maximum value except that of HD. Tables 2 and 3 show the results of biased random walk (BRW) compared with the mass diffusion (MD) and user-based CF (UCF) on* Epinions* and* Friendfeed* data sets, respectively. We can see that BRW algorithm has a higher ranking-accuracy than other algorithms and almost similar accuracy-precision with MD but lower diversity-precision than MD algorithm. It is because the probability of reciprocity links *r*_*L*_ = *L* ^↔^/*L*′ is large in the social network (*Epinions* data set is 45.47% and* Friendfeed* data set is 62.72%), where *L* ^↔^ is the number of bidirectional links and *L*′ is the number of all links in social network. Because it is easier for the random walker to go from one user to another user in social network, the recommendations obtained from social network will be similar among friends.

**Figure 4:**
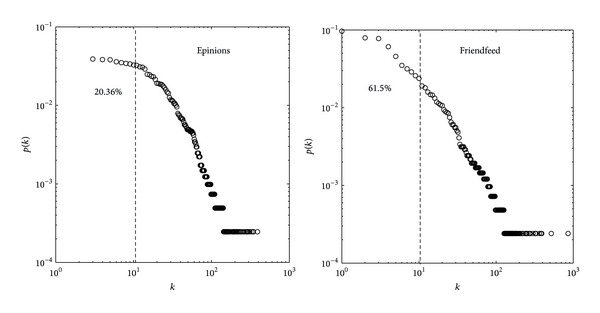
The user degree distribution of training set on* Epinions* and* Friendfeed* data sets.

**Figure 5:**
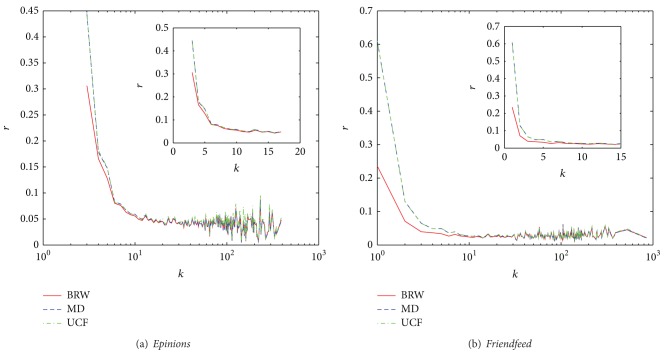
Ranking score values venus degree *k* on* Epinions* and* Friendfeed* data sets (color online). The red line, blue line, and green line indicate the performance of BRW, MD, and UCF, respectively. The inset figure amplifies that ranking score versus the degree of users from 1 to 15.

Generally speaking, the small degree users are the vast majority in the systems (Figure 4 shows the use degree distribution in the training set on* Epinions* and* Friendfeed* data sets. We find that there are 23.06% and 61.5% users with degrees smaller than 10 on* Epinions* and* Friendfeed* data sets, resp.). That is to say, increasing the small degree users' performance could result in performance improvement of the whole system. In Figure 5, we show the effect of user degrees that is in the training set versus ranking score. From the figure we can see that the MD and UCF almost have the same ability for small degree users and our method has better performance than MD and UCF algorithm. Meanwhile, it can be seen that our method considering the social interest into the recommender system has a better performance for both larger and smaller degree users. In other words, it can alleviate the user cold-start problem.

## 6. Conclusion and Discussion

In a real online recommender system, for new users or users with less collections, it is difficult to obtain recommendations because of lack of enough information. However, if they are active in the social network, the system can obtain the recommendations from their friends or social leaders. In this way, the social networks can help us to solve the user cold-start problem.

In this paper, we proposed a recommendation algorithm via biased random walk on a two-layer coupled network: user-object bipartite network and user-user social network. Experiment results on two real data sets indicate that social interest and user's preference can be combined together in a delicate way to improve the accuracy metric of recommendation systems. Compared with two other baseline algorithms, our algorithm achieves the best precision measure and has the best ability of accurately recommending objects to the small degree users, effectively alleviating the user cold-start problem.

This paper only provides a simple method to incorporate the social interest into the recommender systems by random walk on coupled social-information network, while a couple of issues remain open for future study. (i) The structure and evolution of coupled social networks are still unclear to us, but we believe they will be helpful for designing effective recommendation algorithms. (ii) The current algorithm assumes that a random walker goes to his friend on social network and his collected objects on bipartite network with the same probability; we conjecture that an appropriately adjusted weight assignment will further improve the algorithmic performance.

## Figures and Tables

**Table 1:** Properties of the tested data sets.

Data sets	Users	Objects	Collecting links	Social links	Sparsity
*Epinions *	4,066	7,649	154,122	217,017	5 × 10^−3^
*Friendfeed *	4,148	5,700	96,942	386,804	4.1 × 10^−3^

**Table 2:** Algorithmic performance for *Epinions* data set with recommendation list *L* = 20.

Method	*r*	Precision	Recall	*F*-measure	HD
MD	0.172	0.036	0.099	0.046	**0.673**
UCF	0.186	0.033	0.090	0.041	0.56
RW	**0.171**	**0.036**	**0.1**	**0.046**	0.652

**Table 3:** Algorithmic performance for *Friendfeed* data set with recommendation list *L* = 20.

Method	*r*	Precision	Recall	*F*-measure	HD
MD	0.116	0.03	0.140	0.041	**0.9405**
UCF	0.12	0.029	0.0902	0.0386	0.8772
RW	**0.108**	**0.03**	**0.141**	**0.041**	0.9250
